# Two Souls, One Birth: The Vaginal Delivery of Parapagus Conjoined Twins

**DOI:** 10.7759/cureus.81997

**Published:** 2025-04-10

**Authors:** Anu Berwal, Shiwali Saharan

**Affiliations:** 1 Department of Obstetrics and Gynecology, Maharaja Agrasen Medical College, Agroha, IND

**Keywords:** conjoined twins, parapagus twins, siamese twins, unbooked pregnancy, vaginal delivery

## Abstract

Conjoined twins (CTs) are a rare occurrence. A timely prenatal diagnosis of this anomaly by ultrasonography is crucial for counseling regarding various management options like pregnancy termination, selective feticide, or postdelivery surgical separation. However, some cases can still remain undetected until birth. We report the case of a grand multipara in her 30s who came to our labor room at 28 weeks of gestation, as an unbooked case and had vaginal delivery of dithoracic parapagus CTs, weighing 2,000 g. Although cesarean section is considered the preferred mode of delivery for CTs, undiagnosed cases presenting directly at birth, as in our instance, pose significant challenges. In extreme cases, destructive procedures may become necessary. Such cases highlight the need for preparedness during delivery. Sharing the experiences of vaginal deliveries of CTs might help in exploring the optimal approaches for trial of vaginal birth in specific scenarios.

## Introduction

Conjoined twins (CTs), popularly known as Siamese twins, develop when two fetuses of monozygotic origin are joined together at any part of the body. They derived their name from the Greek word "pagus," which means "fused together." They are rare, with a reported prevalence of 1.47 per 100,000 births [1]. The origin of CT is uncertain, with two opposing theories described in the literature. The first one, known as the "fission theory," suggests their development from the incomplete fission of a single embryonic disc, occurring approximately 13-15 days after fertilization. While the "fusion theory" proposes that the fertilized ovum splits completely into two embryonic discs, their close proximity leads to secondary fusion as the embryos grow [2].

The survival of CT depends on the type of CT, the sharing of organs, and timely and appropriate surgical or nonsurgical management. The rate of stillbirth is high and is estimated to be around 60% [3]. Given such high morbidity and mortality associated with conjoined twinning, early prenatal diagnosis is crucial for informed decision-making.

Despite advancements in ultrasonography, undetected cases still occur. Such undiagnosed cases pose significant risks of perinatal and maternal morbidity. While cesarean section is typically the preferred mode of delivery, vaginal delivery may be considered in specific scenarios, such as preterm, low birth weight, or demised twins. Due to the rarity of CT deliveries, particularly the vaginal births, each case warrants detailed documentation to enrich the medical literature. Here, we present a case of dithoracic parapagus CTs, diagnosed at birth and delivered vaginally. This report underscores the importance of timely prenatal diagnosis in guiding the delivery plan.

## Case presentation

**Figure 1:**
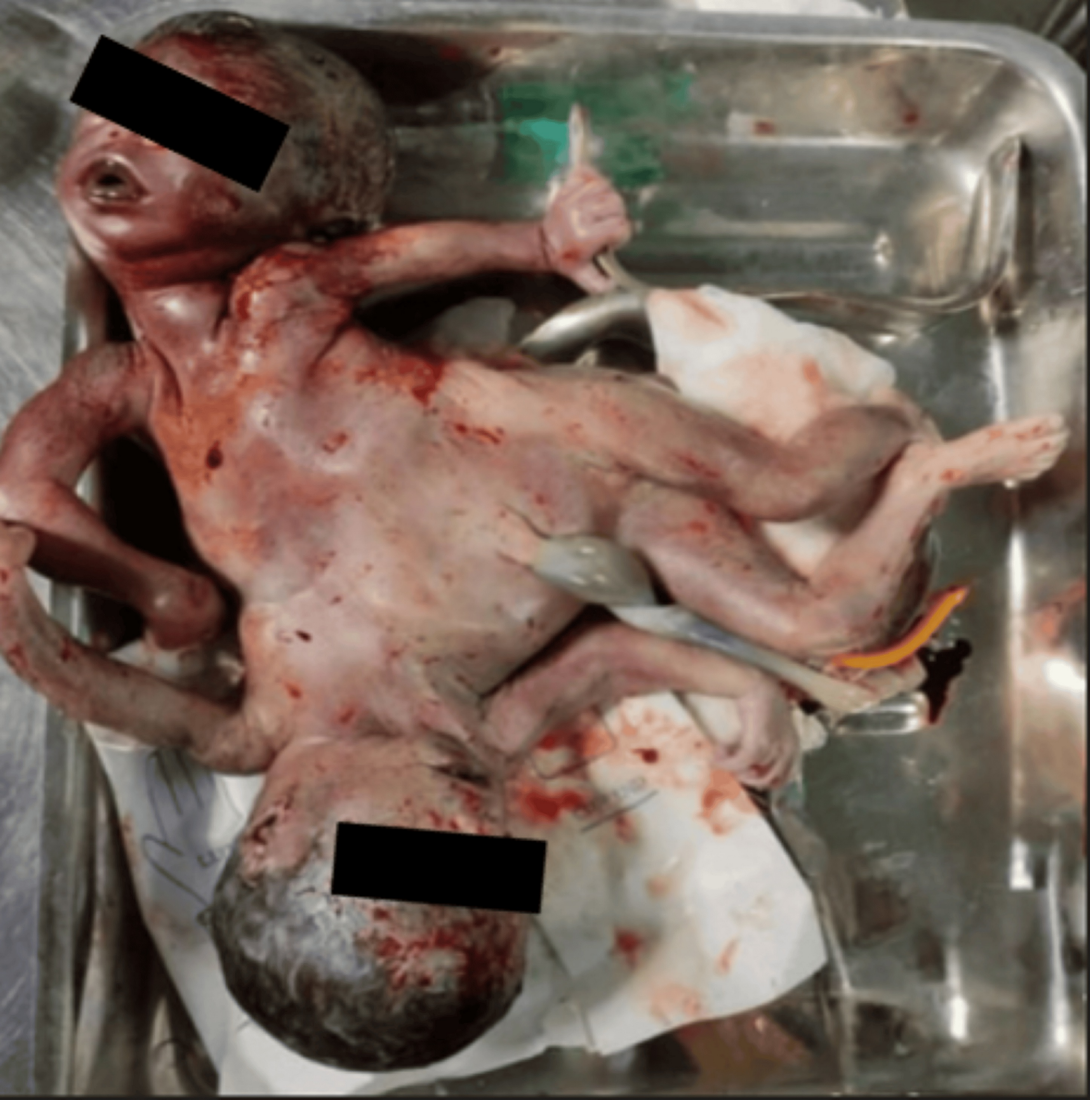
Stillborn dithoracic parapagus conjoined twins

A 37-year-old, unbooked grand multipara with previous five uneventful vaginal deliveries was referred to our tertiary care center at midnight. She was at 28 weeks of gestation and in labor with meconium-stained liquor for the past two hours. She had no family history of twinning and no history of intake of ovulation-inducing drugs. On abdominal examination, the height of the uterus was 32-34 weeks, multiple fetal parts were felt, and fetal heart sounds could not be localized. Her cervix was fully dilated, the vertex was at +2 station, membranes were absent with meconium-stained liquor, and the pelvis was average gynecoid. All routine investigations were sent. The patient was shifted to the delivery table and encouraged to bear down. The head, along with both arms, was delivered spontaneously, after which there was an arrest of further descent. On examination, the hand of twin B was felt alongside the trunk of twin A, which could not be pushed up. On tracing twin A, surprisingly, the ribcage of the second twin was felt in continuity. Emergent sonography was not available in our institute at that hour. Keeping in mind the possibility of CTs which were dead, preterm, and being delivered by a grand multipara with spacious pelvis, trial of vaginal delivery was given after consent. Blood products were arranged, and the operating theater staff were preinformed. A liberal right mediolateral episiotomy of length approximately 5 cm was given. Patient was asked to bear down, and twin A was delivered by traction, along with twin B. Confirming to our suspicion, dead dithoracic parapagus female CTs with a combined weight of 2,000 g were delivered. They were united laterally over the abdomen and pelvis, with separate thoraces. There were four arms and two legs (Figure 1). The placenta was monochorionic monoamniotic with a common three-vesseled umbilical cord. No cervicovaginal or perineal tears were noted. The parents refused fetal autopsy or radiological examination. The patient had an uneventful postpartum period and was discharged on the second day.

## Discussion

CTs are a fascinating phenomenon, with the earliest case described in the Neolithic period [2]. The term "Siamese twins" originated from the famous CTs born in Siam (modern-day Thailand) in 1811. These twins are always monochorionic monoamniotic. Although CTs are more frequently female with an estimated ratio of 3:1, parapagus twins have a notable male preponderance [4,5]. However, in our case, female parapagus twins were born.

Various classifications have been proposed to date for CT, including the simplified and commonly used one given by Kaufman [4] and Spencer [6]. The latest classification described in the International Clearinghouse for Birth Defects Surveillance and Research (2011) is given in Table 1 [1].

**Table 1:** Anatomical classification of CTs CT: conjoined twin Source: [1]

Types	Definitions
Cephalopagus	Two faces; bodies joined from the top of the head to the umbilicus
Thoracopagus	Joined face-to-face from the upper thorax to the upper part of the abdomen and always involves the heart
Omphalopagus	Fusion includes the umbilicus region frequently at the lower thorax, but never the heart
Ischiopagus	Union usually includes the lower abdomen and duplicated fused pelvic bones, and external genitalia and anus are always involved
Parapagus	Laterally joined, regularly share the pelvis. Varieties of parapagus CT are parapagus dithoracic (separated thoraces), parapagus dicephalus (one trunk two separate heads), and parapagus diprosopus (one trunk, one head, and two faces)
Craniopagus	Joined by the skull, share meninges but rarely the brain surface, and do not include the face and trunk
Pyopagus	Dorsally fused sharing the perineal and sacrococcygeal areas and has only one anus but two rectums
Rachipagus	Dorsally fused, and the defect may involve the dorsolumbar vertebral column and rarely the cervical vertebrae and the occipital bone
Other symmetrical	Includes CT that some authors classify differently and also a variety of rare types of symmetrical CT
Asymmetric	Parasitic CT and fetus in fetu

Thoracopagus CT represents the largest number of cases reported (42%). The second most common are parapagus dicephalus (11.5%), followed by omphalopagus (5.5%), cephalopagus (5.5%), parasitic (3.9%), craniopagus (3.4%), parapagus diprosopus (2.9%), ischiopagus (1.8%), rachipagus (1%), pygopagus (1%), and unspecified (21.4%) [1].

Although no radiological investigation or autopsy was conducted in our case, literature suggests that parapagus twins often have variable sharing of organs (Table 2). Complex congenital anomalies are also described, including severe heart malformations, defects of laterality such as situs inversus, asplenia, anomalous lungs, diaphragmatic defects, cleft lip/palate, and anencephaly [7-10].

**Table 2:** Organ sharing in parapagus conjoined twins

Organ/organ system	Extent of sharing
Lungs	Two sets, which may be underdeveloped or anomalous [7]
Heart	Varies from two separate hearts in some dicephalus tripus to either a single compound or normal one in diprosopus [8]
Gallbladder, liver, and pancreas	Frequently shared [8,11]
Gastrointestinal tract	Sharing varies from only the caudal half in some dicephalus to the entire tract in diprosopus [8]
Genitourinary tract	Frequently shared [11]

The antenatal diagnosis of CTs can be challenging, particularly when imaging studies are not performed. As per National Family Health Survey 5 (2019-21), the percentage of women with at least one ultrasound during pregnancy was approximately 78% in India and 88.3% in our state [12,13]. While this indicates a fairly average antenatal care coverage, it also suggests that a significant proportion of cases are still rendered unbooked or inadequately followed up. In our area, missed antenatal care is usually due to illiteracy, unawareness, or limited healthcare access in remote places. Our patient, however, additionally, did not consider it necessary due to past uneventful pregnancies.

In our institute, if a congenital malformation is diagnosed during labor, an emergent sonographic evaluation is performed if feasible to assess severity. The attending obstetrician provides a detailed explanation to the family regarding the anomaly, potential neonatal outcomes, and management options. A multidisciplinary team, including a neonatologist and pediatric surgeon, if available, is consulted. In cases of severe malformations incompatible with extrauterine life, cesarean section is deferred unless there is an obstetrical indication. All discussions, including prognosis and delivery mode, are documented, and informed consent is obtained. The neonatal team is informed in advance to ensure preparedness for immediate postnatal care. This protocol ensures standardized and ethical decision-making.

Early detection of CTs relies on vigilant sonology. Routine first-trimester and mid-second-trimester scans done at 11-13 + 6 weeks and 18-24 weeks play a crucial role in identifying the anomalies in a timely manner [14,15]. However, CTs can be diagnosed as early as the eighth week of gestation by transvaginal ultrasonography as a bifid appearance of the fetal pole [16]. Other specific ultrasonographic criteria include inseparable fetal bodies, lack of change in relative positions of fetal heads and bodies, heads positioned at the same level and body plane, unusual proximity or hyperextension of spines, and close proximity of limbs [17]. Three-dimensional Doppler ultrasonography and MRI have proven to be an effective tool, aiding families in decision-making regarding pregnancy continuation or termination.

Cesarean section is the preferred mode of delivery for CTs to minimize risks to both mother and fetuses. However, undiagnosed cases like ours may present during vaginal birth and pose significant challenges. While compressible fetal tissues can occasionally allow vaginal delivery, there is a risk of uterine rupture, cervicovaginal trauma, or postpartum hemorrhage. Vaginal delivery may be considered in cases of low birth weight, preterm, and nonviable fetuses. Literature describes several maneuvers to facilitate vaginal delivery, but in extreme cases, destructive procedures may become necessary [18].

Advances in imaging technology, prenatal care, and surgical separation techniques have significantly improved the outcomes of CT, particularly where separation is deemed feasible. In the context of parapagus twins, survival is further complicated by the degree of anatomical fusion and the associated organ anomalies. Multidisciplinary teams including pediatric surgeons, anesthesiologists, and neonatologists are crucial in managing such cases.

No national or international guidelines exist for managing CTs diagnosed during labor. Each patient needs an individualized approach. We propose the following protocol for such scenarios in Table 3.

**Table 3:** A proposed protocol for managing conjoined twins diagnosed during labor

Emergent sonographic evaluation	Assess severity, viability, and potential neonatal prognosis
Multidisciplinary consultation	Involve a neonatologist and pediatric surgeon
Counseling and documentation	Explain diagnosis, prognosis, and management options to the family; obtain informed consent
Delivery planning: decision-making should be case-based, prioritizing maternal well-being	Live, viable fetuses or dead fetuses near term: proceed for cesarean section; Dead, nonviable, small babies, spacious maternal pelvis: trial of vaginal birth, unless maternal indication arises for cesarean section; Obstructed vaginal delivery: consider cesarean section after discussion (destructive procedures can be tried if skilled obstetrician available)
Neonatal preparedness	Inform the neonatal team for immediate postnatal care

## Conclusions

Early detection of CTs is crucial to enable timely counseling and offering the option of pregnancy termination. While antenatal diagnostic strategies have improved significantly over the years, factors such as suboptimal imaging conditions, late booking for antenatal care, or misinterpretation of ultrasound findings may contribute to missed diagnoses. Such undiagnosed cases limit birth preparedness and increase both maternal and perinatal risks. Our case highlights the need for prenatal screening and preparedness for unexpected challenges during delivery, ensuring optimal maternal-fetal outcomes. Given the rarity of vaginal deliveries of CTs, compiling and sharing individual experiences is essential for enhancing collective knowledge and improving future management strategies.
